# Motor function may differentiate attention deficit hyperactivity disorder from early onset bipolar disorder

**DOI:** 10.1186/1744-9081-5-47

**Published:** 2009-12-10

**Authors:** Anne H Udal, Ulrik F Malt, Hans Lövdahl, Bente Gjaerum, Are H Pripp, Berit Groholt

**Affiliations:** 1 Department of Child and Adolescent Mental Health, Sörlandet Hospital, PO Box 605 4809 Arendal, Norway; 2 Department of Psychosomatic Medicine, Rikshospitalet University Hospital, 0027 Oslo, Norway; 3 University of Oslo, PO Box 1081 0317 Oslo, Norway; 4 Department of Mental Health, Sörlandet Hospital, PO Box 605, 4809 Arendal, Norway; 5 Centre for Child and Adolescent Mental Health Eastern and Southern Norway PO Box 4623, 0405 Oslo, Norway; 6 Department of Biostatistics, Rikshospitalet University Hospital, 0027 Oslo, Norway; 7 Department of Child and Adolescent Psychiatry, Ullevaal University Hospital, PO Box 26, 0319 Oslo, Norway

## Abstract

**Background:**

Differentiating between bipolar spectrum disorder (BD) and attention deficit hyperactivity disorder (ADHD) in childhood and adolescence is difficult because the clinical presentation is influenced by ongoing neural development, causing considerable symptom overlap. Motor problems and neurological soft signs have been associated with ADHD for decades. Little is known about motor skills in BD. Here we assess the diagnostic accuracy of neuromotor deviations in differentiating ADHD from BD in clinical practice. We also investigate if these deviations exist in concurrent ADHD and BD, thus indicating true comorbidity

**Methods:**

64 patients 6-18 years (31 girls, 33 boys) fulfilling the diagnostic criteria of BD, ADHD combined subtype (ADHD-C) or comorbid BD and ADHD-C, were compared using an age-standardized neuromotor test; NUBU. Categorical variables were analyzed using cross table with two-tailed chi square test or Fisher's exact test when appropriate. Continuous variables were analyzed by Kruskal-Wallis test and, if significant, Mann-Whitney U test and ROC plots.

**Results:**

The ADHD-C group and the comorbid ADHD-C and BD group both showed significantly more neurological soft signs (p less than 0.01) and lower mean static coordination percentile (p less than 0.01) than the BD group. The positive predictive value of NUBU in the diagnosis of ADHD-C with or without concurrent BD was 89% (80-95) for total soft signs and 87% (79-95) for static coordination below the 7.5 percentile.

**Conclusion:**

An age-standardized neuromotor test battery may promote diagnostic accuracy in differentiating ADHD from BD in clinical practice, and help evaluating whether symptoms of ADHD in children who have BD reflect symptom overlap or real comorbidity. This may have important implications for everyday diagnostic work.

## Background

The frequent co-occurrence of BD and ADHD in children has been a matter of much discussion and research [1]. Still, concurrent diagnosis of BD and ADHD remains controversial. There is a considerable symptom overlap between the diagnostic criteria of ADHD and mania. Attention deficits are often found in BD [2] and affective episodes are common in ADHD [3]; manic symptoms in children may be a marker of severe psychopathology rather than of a specific diagnosis [4]. Ongoing brain development influences the clinical presentation and makes it even more difficult to separate the two disorders. Neuroimaging studies indicate biological differences between the disorders [5], but these methods are not useful in clinical practice. It is therefore a need for clinical useful signs to support our descriptive diagnoses.

### Neuromotor problems in ADHD

Motor skill problems and neurological soft signs have been associated with inattention and behaviour difficulties for decades. Concurrent developmental coordination disorder (DCD) is reported in about 50% of children with ADHD [6,7], mainly correlated to combined or inattentive subtypes [7,8]. The earlier minimal brain dysfunction term included soft signs or motor problems as signs of neurological dysfunction [6]. Some authors argue that these signs should be included in the diagnostics of ADHD [9,10]. A review found that movement behaviour in ADHD were described in 49 papers between 1949 and 2002; indicating that movement skill difficulties, poor level of physical fitness and concurrent DCD are frequent in ADHD [11]. Movement skill difficulties often reported are impaired timing [12], impaired hand dexterity/fine motor coordination [13], "motor overflow" and neurological soft signs [10,14,15]. Many of these terms indicate difficulties inhibiting motor responses, which is a consistent finding in ADHD and seemingly varies with task complexity [16]. Balance problems are also often reported in ADHD and are thought to be of cerebellar origin [8,17-19]. Cerebellar abnormalities may also be involved in other motor problems in ADHD. A meta-analysis of structural neuroimaging studies in ADHD found prominent reductions in cerebellum [20], and a recent diffusion tensor study in youths with ADHD showed increased fractional anisotropy in white matter pathways connecting prefrontal and parietal-occipital areas with striatum and cerebellum [21]. Deficient signalling of the prefrontal cortex (PFC) by parietal cortex, basal ganglia and cerebellum may underlie the poor cognitive, motor and behaviour adjustment in ADHD [22]. The motor problems in ADHD may thus be due to poor adjustment in different contexts rather than primary motor deficits, explaining the variability in performance often reported in the ADHD literature.

### Neuromotor problems in BD

There are few reports on motor problems in BD. In two studies, patients with BD were impaired on sequential motor performance consistent with the frequent reported impaired attention set-shifting in BD [23,24]. In contrast, the ADHD subjects were impaired on repetitive motor performance, indicating motor inhibition problems [24]. Another study revealed fine motor skills impairment linked to depressive episodes only [25]. A review paper found significantly more soft signs in schizophrenia and mood disorders versus when compared to normal controls. Poor stereognosis and rhythm tapping were more prevalent in mood disorders than in schizophrenia. Poor diadochokinesia, tandem walk, finger-thumb opposition and articulation was more prevalent in schizophrenia than in mood disorders [26]. Neuroimaging studies in BD suggest abnormalities in a ventromedial PFC-amygdala network regulating mood, and a linked dorsolateral PFC-subcortical network modulating cognition [27]. Decreased amygdala volume is a consistent finding in youths but not in adults with BD [28]. The PFC deficits appears to emerge during adolescence [29]. A hypothesis is that primary amygdala or anterior cingulate deficits causes stress-induced volumetric abnormalities in PFC and other regions [27]. Theoretically these findings should not indicate motor problems in BD. However, in mainly adult BD patients with multiple episodes more structural abnormalities are found, including ventricle enlargement [30], white matter hyperintensities and cerebellar abnormalities [19]. The latter was associated with the number of depressive episodes, and might give rise to motor adjustment symptoms.

### The purpose of this study was twofold

1. Investigate if prevalence of motor deviations differs between youths with ADHD and BD. 2. Investigate if these deviations also exist in concurrent ADHD and BD, thus indicating true comorbidity. A priori hypotheses: 1. Neuromotor deviations are a common feature of ADHD but not of BD. 2. Neuromotor deviations occurring in concurrent ADHD and BD are indicative of true comorbidity.

## Methods

### Inclusion criteria

Inclusion criteria were ADHD combined subtype (ADHD-C), BD and concurrent ADHD-C and BD (ADHD-C+BD). We included ADHD-C only, because data supporting the validity of other subtypes of ADHD is scarce [31]. Those with BD and ADHD-C were classified as ADHD-C+ BD. Those with BD and other subtypes of ADHD were classified as BD only. BD was defined as BP-I or BP-II according to DSM-IV [32], or BP-NOS according to the Course of Bipolar Youth criteria [33]: "A minimum of elated mood plus 2 associated DSM-IV symptoms, or irritable mood plus 3 DSM-IV associated symptoms, along with a change in the level of functioning, duration of a minimum of 4 hours within a 24-hour period, and at least 4 cumulative lifetime days meeting the criteria". This is a possible bipolar category; 25% percent of children and adolescent fulfilling these criteria are shown to convert into BP-I or BP-II in 2 years [33]. Patients with longer hypomania episodes without depressive episodes and patients with cyclothymic disorder according to DSM-IV were also categorized as BP-NOS.

### Exclusion criteria

Mental retardation according to DSM-IV or sequelae of brain injury.

### Procedure

Subjects were recruited from a general child and adolescent psychiatry outpatient unit, mainly from one Norwegian community with approximately 25,000 persons <18 years. Inclusion period was December 2004 to April 2008, 1267 subjects (586 females) were referred to the unit in this period. In addition ten subjects were referred from other child and adolescent psychiatry units because of this study. Whenever bipolar disorder was suspected (n = 208) by any of the employees, the patients were evaluated for further assessment. Finally 172 subjects were interviewed by the Kiddie-Schedule for Affective Disorders and Schizophrenia Present and Lifetime version (KSADS)[34]. Caregiver(s) and children > 8 years were interviewed separately by a psychiatrist trained by the child and adolescent psychiatrist responsible for translating and coursing of KSADS in Norway. The taped interviews, supplied with condensed anamnestic information, were validated by a child and adolescent psychiatrist. Interrater agreement (kappa) was 1.0 on bipolar disorder and 0.87 on ADHD-C.

Most of the patients with ADHD-C were recruited from subjects with suspected BD, who fulfilled the ADHD-C criteria only according to the KSADS interview. Ten additional patients with suspected ADHD-C only were selected from patients at the unit to optimize the age, gender and IQ match between the groups. These went through the same diagnostic assessments as the other participants.

In addition to diagnoses of ADHD-C and BD, other diagnoses and background information were recorded from KSADS. All subjects completed an IQ-test (the Wechsler Intelligence Scale for Children-III Norwegian version or the Wechsler Adult Intelligence Scale III Norwegian version, supplied with the Vineland Adaptive Behaviour Scales when required). Further information was obtained from school (Teacher Behaviour Rating Scale [35]), teachers evaluation and in most cases school observation). Subjects received standard treatment; this was not a part of the study but was recorded as clinical information. The final diagnosis was based on KSADS and all available information including response to medication within one month after inclusion, in a discussion with the main researcher and an experienced child psychiatrist (last author).

### Subjects

Sixty-six subjects were initially included. Two female BP-I patients were later excluded; one because of drug abuse during testing and one because of possible perinatal brain injury. Sixty-four subjects grouped as ADHD-C, ADHD-C+BD or BD were finally included in the study. Six patients with concurrent ADHD-C and BP-NOS symptoms no longer satisfied the criteria for BP-NOS after stimulant medication; these were reclassified as ADHD-C only. One patient with BP-I successfully treated by a mood stabilizer, switched to mania when adding a stimulant to treat concurrent ADHD symptoms, this patient was therefore classified as BP-I only.

Twenty-six fulfilled the criteria for ADHD-C only, mean age 13.2 years (S.D. = 3.8 years), mean total IQ 91.4 (S.D. = 10.9), 11 females. Fifteen fulfilled the criteria for both ADHD-C and BD (BP-I = 3, BP-II = 4, BP-NOS = 8), mean age 14.0 years (S.D. = 3.6 years), mean total IQ 90.1 (S.D. = 14.7), females 7. Twenty-three fulfilled the criteria of BD (BP-I = 8, BP-II = 7, BP-NOS = 8), mean age 13.8 years (S.D. = 3.8 years), mean total IQ = 95.1 (S.D. = 13.3), females 13. Differences in age and total IQ were non-significant.

### Motor examination

The Neuromotor examination for children and adolescents 4-16 years (NUBU) was used [36]. It includes a revised version of the neurological soft sign test from the Isle of Wight Study [37] and motor tests developed from the Oseretsky's test [38]. NUBU is age standardized in a recent study of 272 representative Norwegian children and adolescents without known developmental problems [36]. A soft neurological sign refers to a minor neurological finding, indicating neurological dysfunction depending on age. The soft sign tests in NUBU are the same for all ages; deviations are defined as performance inferior to 85% of the normally developing children. The motor test covers five different domains, each with ten age standardized items. The norms are based on a Rasch model and expressed in terms of age equivalents and percentiles in the range 3.5 to 18 years. In the normative sample, Rasch person reliability was 0.94 and Rasch interrater reliability was 0.99 (Harald Janson, personal communication).

### NUBU 4-18 Soft signs; age for all evaluation criteria mastered

1. Total soft signs (summarized test 2-12)

2. 20 jumps on one foot (6 years)

3. Fingertip touch with open (5 years) and closed eyes (6 years)

4. Oculomotor function; coordinated eye movements without head following (7 years)

5. Stretch arms forward for 20 seconds without involuntary or abnormal movements (8 years)

6. Walking heel-toe on line for 20 paces (8 years)

7. Speech; pronunciation and comprehension (8 years)

8. Standing on one foot for 20 seconds (8 years)

9. Diadochokinesis (8 years)

10. Cutting a paper circle (8 years)

11. Fog's test: Walking on lateral sides of feet (11-12 years)

12. Finger opposition (15-16 years)

### NUBU 4-18 Motor tests; ten age standardized items of each test

1. Total motor age (mean value of test 2-6)

2. Static coordination (postural control in different positions without moving the feet)

3. Hand-eye coordination (hand-eye coordination and ball tests)

4. Dynamic coordination (postural control in different moving positions)

5. Motor tempo (tempo and precision in hand- and postural movements)

6. Simultaneous movement (motor coordination and sustained rhythm in simultaneous motion)

We used the percentile scores in our group comparisons. The NUBU testing was performed by a psychiatrist trained by the authors of NUBU and by two physiotherapists with special competence in child and adolescent psychiatry, supported by a detailed manual with DVD demonstration of the NUBU tests. Interrater reliability was established by two of the investigators, testing 6 patients together. For motor percentile test, Intraclass Correlation Coefficient range was 0.91-1.00 (single measure). Soft sign tests kappa measure of agreement range was 0.57 - 1.00 (kappa). Inclusion date and test date were not significantly correlated with total soft sign deviations or total motor age.

We attempted to do the test in euthymic and drug free patients. This was impossible in a minority of the subjects because of severity of symptoms (mania or psychosis; n = 9), these were assessed using mood stabilizers. All other medication was discontinued for a minimum of five times the elimination half-life before testing. We had to retest the first included patients (n = 12) after two years because the NUBU scoring algorithms were slightly changed. Missing data: One did not do the dynamic coordination subtest because of discus prolapse and one did not co-operate in the tempo subtest. There were no missing data in the soft sign tests.

### Self reported motor problems

The KSADS does not include the diagnostics of DCD, but the introductory part contains questions about motor development and motor difficulties. Answers on these questions were compared with the NUBU findings.

### Statistical analysis

Data were analyzed using the Statistical Package for Social Sciences (SPSS), version 16. Analyzed factors were soft sign deviations and motor age percentiles adjusted for possibly confounders (comorbidity and medication). Categorical variables were analyzed using cross table with two-tailed chi square test (with Yates Continuity Correction) or Fisher's exact test when appropriate. Continuous variables were analyzed by Kruskal-Wallis test and if significant, pairwise comparisons using Mann-Whitney U test. ROC curves were used to decide cut off value for presence of ADHD-C diagnosis and corresponding sensitivity, specificity and predictive values. Reported motor problems (from the introductory part of KSADS) were compared with total motor problems and total soft sign deviations by Spearman correlation.

### Ethics

The protocol was approved by the Regional Committee for Medical Research Ethics of Southern Norway and the Norwegian Social Science Data Services. All children and their caregivers were given verbal and written information. Caregivers gave formal written consent for all children under age 18 years. Children ≥ 12 years gave formal written consent, younger children gave spoken consent. Data collection was mostly incorporated in routine clinical work. Considering the uncertainty of psychiatric diagnosis in childhood and adolescence, all subjects were offered diagnostic reassessment after 2-3 years and after the age of 18.

## Results

### Soft signs

Soft sign deviations and differences between the groups are shown in Table 1. Both the ADHD-C group and the ADHD-C+BD group had significantly more total soft signs than the BD group, also when excluding BP-NOS from the analysis. Deviations in finger opposition were most frequent in all groups, but significantly higher in ADHD-C and ADHD-C+BD than in BD. The most specific sign in ADHD-C was oculomotor function, but the sensitivity of this soft sign was rather low. There were no significant differences between the ADHD-C and ADHD-C+BD groups.

**Table 1:** Soft signs deviations; differences between groups including and excluding BP-NOS

Mean soft sign deviations below the 15^th ^percentile#	A;n = 26 n (%)	AB;n = 15 n (%)	B;n = 23 n (%)	ABx;n = 5 n (%)	Bx;n = 16 n (%)	A vs B [A vs Bx] test value, p #	AB vs B [ABx vs Bx] test value, p #	A vs AB [A vs ABx] test value, p #
**Total**	22 (85%)	11 (73%)	4 (17%)	5 (100%)	3 (19%)	19.53*** [17.84***]	9.67*** [7.50***]	n.s. [n.s.]

**Jump one leg**	16 (62%)	6 (40%)	5 (22%)	0 (0%)	3 (19%)	6.35** [5.70** ]	n.s. [n.s.]	n.s. [Fisher** ]

**Fingertip touch**	5 (19%)	2 (13%)	3 (13%)	1 (20%)	2 (13%)	n.s. [n.s.]	n.s. [n.s.]	n.s. [n.s.]

**Oculomotor**	8 (31%)	2 (13%)	1 (4%)	0 (0%)	0 (0%)	Fisher ** [Fisher**]	n.s. [n.s.]	n.s. [n.s.]

**Arms forward**	5 (19%)	4 (27%)	1 (4%)	1 (20%	0 (0%)	n.s. [n.s.]	n.s. [n.s.]	n.s. [n.s.]

**Walk heel-toe**	7 (27%)	6 (40%)	4 (17%)	2 (40%)	2 (13%)	n.s. [n.s.]	n.s. [n.s.]	n.s. [n.s.]

**Speech**	5 (19%)	3 (20%)	2 (9%)	1 (20%)	1 (6%)	n.s. [n.s.]	n.s. [n.s.]	n.s. [n.s.]

**Stand one leg**	14 (54%)	7 (47%)	7 (30%)	3 (20%)	4 (25%)	n.s. [n.s.]	n.s. [n.s.]	n.s. [n.s.]

**Diadochokinesis**	12 (46%)	5 (33%)	4 (17%)	1 (20%)	4 (25%)	n.s. [n.s.]	n.s. [n.s.]	n.s. [n.s.]

**Cutting circle**	3 (13%)	4 27%)	1 (4%)	1 (20%)	1 (6%)	n.s. [n.s.]	n.s. [n.s.]	n.s. [n.s.]

**Fog's test**	14 (54%)	7 (46%)	7 (30%)	3 (60%)	6 (38%)	n.s. [n.s.]	n.s. [n.s.]	n.s. [n.s.]

**Finger opposition**	22 (85%)	12 (80%)	8 (35%)	3 (60%)	7 (45%)	10.75*** [5.95**]	5.74** [n.s.]	n.s. [n.s.]

A = ADHD-C, B = BD, AB = A+BD, Bx = BD excluding BP-NOS, ABx = A+Bx. # Two-tailed Chi square/Fisher, df = 1, ***p < 0.01, **p < 0.03, *p < 0.05, n.s. = nonsignificant. #Number and percentage refers to those with performance inferior to 85% of the normally developing children.

### Motor age tests

Median motor age percentiles and differences between the groups are shown in Table 2. The ADHD-C group performed significantly worse than the BD group on all tests except tempo. The ADHD-C+BD group performed significantly worse than the BD group on all tests except dynamic coordination and tempo.

**Table 2:** Median motor percentiles; differences between groups including and excluding BP-NOS.

Motor tests (percentiles) A vs AB vs B #	A n = 26 median	AB n = 15 median	B n = 23 median	ABx n = 5 median	Bx n = 16 median	A vs B [A vs Bx] U,z,p ¤	AB vs B [ABx vs Bx] U,z,p ¤	A vs AB [A vs ABx] ¤
Total Age χ^2 ^(n = 64) = 11.53***	19 1-96	6 1-88	88 18-100	6 1-75	73 18-100	93.5, -4.1*** [83.0, -3.24***]	50.5, -3.65*** [14.5, -2.11*]	n.s. [n.s.]

**Static Coordination** χ^2 ^(n = 64) = 12.66***	4 1-72	1 1-99	63 1-100	1 1-6	70.5 1-100	114.0, -3.75*** [93.5, -3.03***]	48.5, -3.80*** [10.0, -2.53**]	n.s. [n.s.]

**Eye Hand Coordination** χ^2 ^(n = 64) = 4.77***	18.5 1-99	26 1-94	88 75-97	89 1-94	76 2-100	155.0, -2.89*** [120.0, -2.29**]	89.9, -2.50** [n.s.]	n.s. [n.s.]

**Dynamic Coordination** χ^2 ^(n = 63) = 6.01*	60.5 1-99	85 1-99	87.5 1-99	88 75-97	88 1-100	163.0, -2.54** [111.5, -2.27**]	n.s. [n.s.]	n.s. [n.s.]

**Tempo** **n.s**.	42 1-100	83 1-100	88 1-95	83 1-96	87.5 1-100	n.s. [n.s.]	n.s. [n.s.]	n.s. [n.s.]

**Simultaneous** χ^2 ^(n = 64) = 2.23**	55 1-100	13 1-95	83 1-100	28 1-94	66 1-100	198.0, -2.01* [n.s.]	89.0, -2.51** [n.s.].	n.s. [n.s.]

A = ADHD-C, AB = ADHD with BD, B = BD, ABx = ADHD with BD excluding BP-NOS, Bx = BD excluding BP-NOS

# Kruskal-Wallis Test (df = 2), ¤ Mann-Whitney U test (df = 1), ***p < 0.01; **p < 0.03, *p < 0.05, n.s = nonsignificant

When the BP-NOS patients were excluded from the analysis (renaming the BD groups BDx and ADHD-C+BDx), the ADHD-C group performed significantly worse than the BDx group on all tests except tempo and simultaneous movement. Median motor age percentiles remained significantly lower in the ADHD-C+BDx group compared to the BDx group on total motor age and static coordination.

There were no significant differences between the ADHD-C and the ADHD-C+BD or ADHD-C+Bx groups.

### Diagnostic accuracy

Sensitivity, specificity, and predictive values of soft signs in predicting ADHD-C diagnosis (with or without BD) are shown in Table 3.

**Table 3:** Sensitivity, specificity, PPV and NPV for soft signs in predicting ADHD-C (N = 41), versus no ADHD-C (N = 23).

Soft signs	Sens. % (C.I.)	Spes, % (C.I.)	PPV,% (C.I.)	NPV, % (C.I.)
**Total**	81 (72-86)	83 (68-92)	89 (80-95)	70 (58-79)

**Jump on one leg**	54 (45-60)	78 (62-90)	82 (68-91)	49 (39-56)

**Fingertip touch**	17 (10-22)	87 (75-95)	70 (42-89)	32 (32-41)

**Oculomotor**	24 (18-26)	96 (83-99)	91 (65-98)	41 (36-42)

**Arms forward**	22 (15-24)	96 (83-99)	90 (63-98)	41 (36-42)

**Walk heel-toe**	32 (24-37)	83 (68-93)	77 (57-90)	40 (33-45)

**Speech**	20 (13-23)	91 (79-98)	80 (52-94)	39 (34-42)

**Stand on one leg**	51 (42-59)	70 (53-85)	75 (62-86)	44 (34-53)

**Diadochokinesis**	42 (33-47)	83 (67-93)	81 (65-92)	44 (36-59)

**Cutting circle**	17 (11-19)	98 (85-99)	88 (56-98)	39 (35-41)

**Fog's test**	51 (42-59)	70 (53-83)	75 (62-86)	44 (34-53)

**Finger opposition**	83 (74-89)	68 (52-80)	83 (74-89)	68 (52-80)

sens = sensitivity, spes. = specificity, PPV = positive predictive value,

NPV = negative predictive value. 95% C.I. = 95% confidence interval.

Using ROC curves we found that a static coordination percentile below or equal to 7.5 was the best motor age predictor for ADHD-C diagnosis (with or without BD). The sensitivity of the other motor tests was too low to be used as signs of ADHD-C (Table 4), although differences between the ADHD-C and no ADHD-C groups were statistically significant in all tests except tempo.

**Table 4:** ROC-analysis of motor percentiles based on ADHD-C versus no ADHD-C

Motor Percentile	**Area under curve, C.I**.	**Cut perc**.	Sens % C.I.	**Spec % C.I**.	**PPV% C.I**.	**NPV % C.I**.
Total motor age***	.836 .737 - .936	14.0	44 37-44	100 88-100	100 85-100	50 44-50

Static coordination**	.820 .703 - .937	7.5	76 67-81	83 67-92	87 79-95	66 53-73

Hand-eye coordination**	.734 .610 - .858	14.5	44 36-49	91 77-98	90 74-97	48 40-51

Dynamic coordination**	.668 .531 - .804	15.5	24 18-26	96 83-99	91 65-98	42 36-43

**Tempo**	.639 .502 - .775	16.5	38 32-41	96 82-99	94 76-99	47 40-49

Simultaneous movement**	.693 .553 - .834	12.0	34 26-39	87 72-95	82 63-94	43 35-47

Area under the curve and suggested cut-off percentiles with and corresponding sensitivity, specificity, positive predictive value and negative predictive value in predicting ADHD-C (N = 41) versus no ADHD-C (N = 23).

C.I. = 95% confidence interval, Cut perc. = cut-off percentile, sens = sensitivity, Spec = specificity, PPV = positive predictive value, NPV = negative predictive value***p < 0.01; **p < 0.03, *p < 0.05 (null hypothesis: true area = 0.5).

### Confounding comorbidity?

Because anxiety disorders may be associated with postural instability [39,40], we compared the differences in comorbidity of these disorders. Concurrent anxiety disorders (lifetime separation anxiety, generalized anxiety disorder, specific phobia, agoraphobia, panic disorder or post traumatic stress disorder) were found in all groups (ADHD-C 27%, ADHD-C+BD 73%, BD 57%, χ^2^(2,n = 64) = 9.14, p = 0.01). These differences persisted when excluding BD-NOS from the analysis (ADHD-C+BD 80%, BD 56%, χ^2 ^(2,n = 47) = 6.47, p = 0.037).

In this study we compared the three groups ADHD-C, BD and comorbid ADHD-C and BD. In the BD group, most subjects reported considerable attention problems; of which ten fulfilled the diagnostic criteria of ADD. Because some studies indicates that motor deficits are particularly prominent in ADD [7], we moved the ten ADD+BD subjects to the ADHD-C+BD group, renaming it the ADHD-C/ADD+BD group (n = 25). The remaining BD was named the BDr group (n = 13). When we repeated the analysis, the same differences remained with few exceptions: The difference between the ADHD-C and the BDr group was now significant only on total soft signs (χ^2^(1,n = 39) = 17.55, p < 0.000) and finger opposition (χ^2^(1,n = 39) = 8.667, p = 0.003). The ADHD-C group performed significantly worse than the ADHD-C/ADD+BD group on total soft signs (χ^2^(1,n = 51) = 6.297, p = 0.012), jump on one leg (χ^2^(1,n = 51) = 5.790, p = 0.016) and finger opposition (χ^2^(1,n = 51) = 3.878, p = 0.049). There was no change in significant differences between the groups on the motor tests.

### Reported motor problems and NUBU

Caregiver-reported motor problems (from KSADS) and total motor age percentile was only weakly negatively correlated (Spearman's rho = -0.259, n = 63, p = 0.027). There were no differences in soft signs between those who reported motor problems and those who did not (44.4% versus 41.3%).

### Confounding medication?

Nine subjects in the BD or ADHD-C+BD groups were on eleven mood stabilizers or antipsychotics, although these were not taken on the test day (four on lamotrigine, two on valproate, three on lithium, and two children on aripiprazole). The medication group had significantly lower total motor age percentile (median = 18 versus 75, U = 51, Z = -2.73, p = 0.006), simultaneous movement percentile (median = 1 versus 85, U = 43, Z = -3.026 p = 0.02) and hand-eye coordination percentile (median = 28 versus 72, U = 70.5, Z = - 2.064, p = 0.038) than the non-medicated BD group. No other significant differences between the medication and the no medication group were found.

## Discussion

To our knowledge, this is the first report comparing motor skills and soft neurological signs in subjects with ADHD and BD.

Firstly, we found that neuromotor deviations may be used as a possible sign of ADHD-C, thus aiding the differential diagnosis of ADHD-C and BD. Secondly, neuromotor deviations in ADHD-C+BD may be due to ADHD-C and thus indicating that ADHD-C in paediatric BD is a phenotypic copy of ADHD-C in non-BD patients.

Including ADD in the ADHD-C+BD group reduced the differences between the ADHD-C/ADD+BD and BD groups, indicating that the attention deficit is associated with BD mainly, and not with concurrent inattentive subtype of ADHD.

Caregiver-reported motor problems were only weakly correlated with neuromotor deviations in this study. We therefore assumed that our findings might be due to ADHD-C and not solely to concurrent DCD, whose diagnostic criteria require reduced motor performance relative to age in daily activities.

The most prevalent soft sign in ADHD-C was deviations in the complex task finger opposition. This test requires motor timing and inhibition, which are consistently found abnormal in ADHD [41]. Although significantly lower, deviant finger opposition was also frequent in BD (Table 1), unrelated to medication. This is consistent with impaired sequential motor performance reported in BD subjects [23,24]. Moreover, some of the attention deficit symptoms in BD may be correlated with ADHD and thus may contribute to the soft signs noted.

Balance problems in ADHD are confirmed in several studies, but are also common in many other disorders, often associated with cerebellar dysfunction. Cerebellar abnormalities have been reported in several neuroimaging studies of ADHD [19,42]. In a study of youths with a bipolar parent, reduced performance on "standing heel-to-toe" and "standing on one foot with eyes open", was interpreted as cerebellar ataxia [43]. These findings may be markers of ADHD as well, because of increased prevalence of ADHD in relatives of BD patients [1]. Other symptoms of cerebellar dysfunction in youths at risk for BD were not confirmed in that study. In our study, the most specific soft signs in ADHD-C were oculomotor problems suggestive of cerebellar dysfunction (Table 1). However, cerebellar dysfunction may also be associated with schizophrenia and perhaps with prolonged excessive anxiety states [19]. In our study, anxiety disorders were most frequent in BD, thus did not contribute to more balance problems in those with ADHD-C diagnosis. A study reported balance problems in social anxiety and in ADHD, but not in other psychiatric disorders in childhood. In that study ADHD was also correlated with both balance problems and hand dexterity problems [39]. This is consistent with our findings; balance problems may be suitable as a marker for ADHD-C versus BD in combination with "cerebellar" soft signs or hand dexterity problems.

Some patients with ADHD-C scored above the 7.5^th ^percentile on static coordination and in the normal range for soft signs. This may be an expression of the variability of performance reported in the ADHD literature suggesting different pathways involved in the disorder [22].

Compared to other studies, we found more motor difficulties in the ADHD group. Firstly, we only included ADHD combined subtype. Secondly; it may be that ADHD patients actually have specific motor deviations, but most test batteries are not specific enough to uncover these. The test battery often used in diagnosing DCD; the Movement Assessment Battery for Children [44], scores dynamic and static balance together, and manual dexterity tasks together; which may account for the different findings. The fact that parent-reported motor problems were not associated with soft signs or motor age percentiles supports the assumption that NUBU might be more suitable to uncover motor deficits in ADHD than certain other test batteries. However, NUBU is a general motor test battery which is not designed for the specific problems often observed in ADHD and can not be used as a diagnostic test alone.

The motor tests in NUBU are time consuming and require special equipment and are therefore cumbersome in everyday clinical work. However, the demonstration of soft signs requires no special equipment and is far less time consuming. Considering the high positive predictive value of total soft signs, their demonstration may add diagnostic accuracy for ADHD.

### Limitations

This study is based on a convenient and small sample, limiting a generalization of the findings.

Because of uncertain validity of the BD diagnosis in young patients, the findings in BD should be interpreted carefully. Including BD-NOS increases the uncertainty. However, the results mainly remained unchanged when excluding BD-NOS from the analysis. The diagnosis ADD also causes problems; in some cases the symptoms may be due to BD, and in other cases they may be due to the inattentive subtype of ADHD. It is also reasonable to presume that the main investigator improved her proficiency diagnostic assessment during the data collection period. These diagnostic uncertainties will be considered in an ongoing follow up study (diagnostic reassessment after 2-3 years and after the age of 18).

A weakness of NUBU is the contribution of subjective judgement in scoring. Thus, the lack of blinding may have contributed to the high prevalence of neuromotor problems among ADHD-C patients in this study. Yet, the highly significant differences between the groups and the high inter-rater reliability between the raters in this study rule out the possibility that bias rating alone explain the results.

## Conclusion

This study indicates that an age standardized neuromotor test battery may contribute to diagnostic accuracy in differentiating between early onset BD and ADHD-C in clinical practice. It may also contribute in evaluating whether ADHD symptoms in children who have BD reflect symptom overlap or real comorbidity. This may have considerable implications for everyday clinical work, because the soft sign tests can be carried out in any child and adolescent unit or family practice. Specific test batteries should be designed to detect the specific motor problems in ADHD[45]. Further studies are needed with a blinded design, larger samples and a more homogenous BD group.

## Abbreviations

ADHD: attention deficit hyperactivity disorder; ADHD-C: attention deficit hyperactivity disorder, combined subtype; ADD: attention deficit hyperactivity disorder, inattentive subtype; BD: Bipolar spectrum disorder; BP-I: Bipolar disorder I; BP-II: Bipolar disorder II; BP-NOS: Bipolar spectrum disorder not other specified; BDx: Bipolar Disorder I or Bipolar disorder II; BDr: BD excluding those with concurrent ADD; NUBU: Neuromotor examination for children 4-16 years; DCD: Development coordination disorder; PFC: Prefrontal cortex; KSADS: The Kiddie-Schedule for Affective Disorders and Schizophrenia Present and Lifetime version; DSM-IV: Diagnostic and Statistical Manual of Mental Disorders, 4th Edition

## Competing interests

All authors except Bente Gjaerum declare that they have no competing interests.

Bente Gjaerum is the senior author of NUBU, and thus declares a financial interest.

## Authors' contributions

AHU is responsible for conception and design, acquisition of data, analysis and interpretation of the data, drafting, revising and approval of the manuscript

BG, UFM and HL are co-responsible for conception and design, critically revising of the manuscript and have given final approval of the version to be published. BG and UM has also contributed to interpretation of the data.

AHP is responsible for statistical analysis and interpretation of the data, critically revising of the manuscript and have given final approval of the version to be published.

BG is responsible for training AHU in assessment and interpretation of NUBU, critical revising of the manuscript and final approval of the version to be published.
